# Loss of *Parp-1 *affects gene expression profile in a genome-wide manner in ES cells and liver cells

**DOI:** 10.1186/1471-2164-8-227

**Published:** 2007-07-10

**Authors:** Hideki Ogino, Tadashige Nozaki, Akemi Gunji, Miho Maeda, Hiroshi Suzuki, Tsutomu Ohta, Yasufumi Murakami, Hitoshi Nakagama, Takashi Sugimura, Mitsuko Masutani

**Affiliations:** 1 ADP-ribosylation in Oncology Project, National Cancer Center Research Institute, 1-1, Tsukiji 5-chome, Chuo-ku, Tokyo 104-0045, Japan; 2 Biochemistry Division, National Cancer Center Research Institute, 1-1, Tsukiji 5-chome, Chuo-ku, Tokyo 104-0045, Japan; 3 Department of Biological Science & Technology, Faculty of Industrial Science & Technology, Tokyo University of Science, 2641, Yamazaki, Noda, Chiba 278-8510, Japan; 4 Chugai Pharmaceutical Co. Ltd., 1-135, Komakado, Gotemba, Shizuoka 412-0038, Japan; 5 Center for Medical Genomics, National Cancer Center Research Institute, 1-1, Tsukiji, 5-chome, Chuo-ku, Tokyo 104-0045, Japan

## Background

Following the publication of the paper 'Loss of *Parp-1* affects gene expression profile in a genome-wide manner in ES cells and liver cells' [1], we found an error in our data.

In the article, we used six replicates of microarray data of wild-type ES cells for comparison with the microarray data of *Parp-1* knockout ES cells. We found that three replicate data were carelessly included in the data for wild-type ES cells. The comparison should have been carried out between three replicates for the *Parp-1*^+/+^ ES cell line, J1, and three replicates for two *Parp-1*^-/-^ ES cell lines, 210–58 and 226–47, respectively.

Therefore, we re-analyzed the data in ES cells according to the same criteria. The consequences of this error are reflected in changes to our results although the conclusions we obtained in the study are not affected.

### Corrected sentences in the Abstract

Here, we demonstrate that of the 9,640 genes analyzed, in *Parp-1^-/-^* ES cells. 3.6% showed altered gene expression. Of these, 2.5% and 1.1% of the genes were down- or up-regulated by 2-fold or greater, respectively, compared with *Parp-1*^+/+^ ES cells (*p *< 0.05).

## Corrected results in the text

### Gene expression profile in *Parp-1^-/-^* ES cells

**Figure 1:**
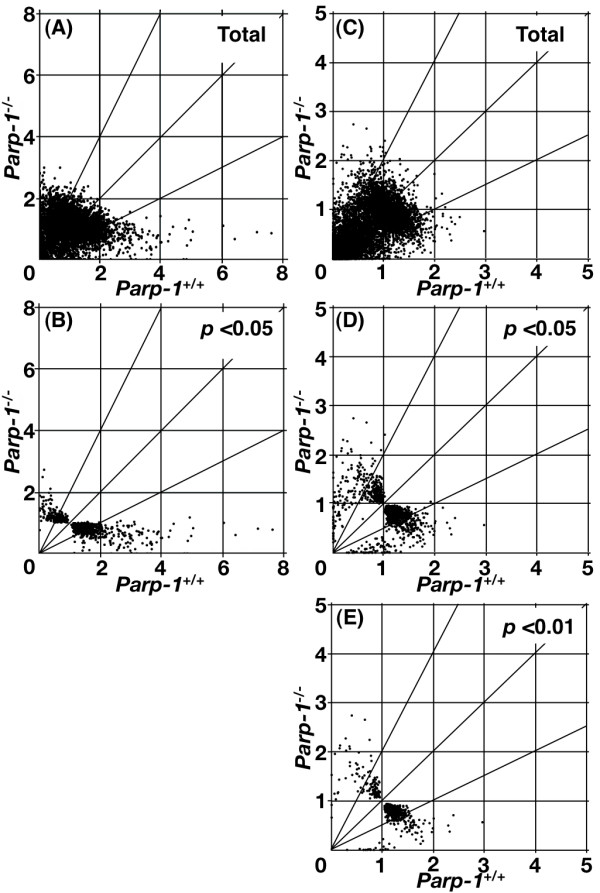
**Effect of *Parp-1 *deficiency on gene expression**. Gene expression data from microarray analyses are plotted for *Parp-1^-/-^*versus wild-type (*Parp-1^+/+^*) ES cell lines (A) & (B). Horizontal and vertical axes represent expression levels normalized for an individual gene. Each point represents normalized expression data for an individual gene. The genes that showed standard deviation greater than 2.0 in the normalized data of both genotypes (A) were excluded and gene lists were constructed with *p *< 0.05 (B). Fig. 1D–F in the original article [1] remains unchanged and is presented as (C) – (E), respectively.

A comparison of the basal gene expression profiles in *Parp-1^-/-^*EScells to their wild-type (*Parp-1^+/+^*) counterparts, is presented in Fig. 1A &1B (corrected) and Table 1 (corrected). We found the expression of (344/9,640) genes, namely 3.6%, was different by at least 2-fold between *Parp-1^-/-^*and *Parp-1^+/+^*ES cells (*p *< 0.05) (Fig. 1B (corrected) and Table 1 (corrected)). Notably, a larger fraction of the genes, being 2.5%(238/9,640), was down-regulated, whereas only 1.1% (106/9,640) of the genes were up-regulated (see Table 1 (corrected)).

**Table 1:** Differential expression of genes between *Parp-1*^+/+ ^and *Parp-1*^-/- ^ES cells, livers, and EFs

	No. of genes				
	
		*Parp-1*^-/- ^<*Parp-1*^+/+^		*Parp-1*^-/- ^> *Parp-1*^+/+^	
			
*p*-value cut off^a^	Total	Total	2-fold or greater	Total	2-fold or greater
ES cells^c^					
Total^b^	9,640	5,065	1,056	4,481	1,520
*p* < 0.05^b^	893	663	238	230	106
					
Livers^d^					
Total^b^	12,353	7,138	1,184	4,860	1,038
*p* < 0.05^b^	1,616	1,190	253	426	158
*p* < 0.01^b^	641	515	100	126	43
					
EFs^e^					
Total	12,357	5,042	707	7,317	501
*p* < 0.05	996	390	216	606	205

^a^Analyzed by One-Way ANOVA (non-parametric test known as the Mann-Whitney U test)

^b^These genes were presented in Fig. 1.

^c^*Parp-1*^+/+ ^ES cell clone, J1, and *Parp-1*^-/- ^ES cell clones, 210–58 and 226–47, were used.

^d^Two mice were used for each genotype.

^e^Three EFs obtained from three embryos were analyzed as triplicate experiments.

**Figure 2:**
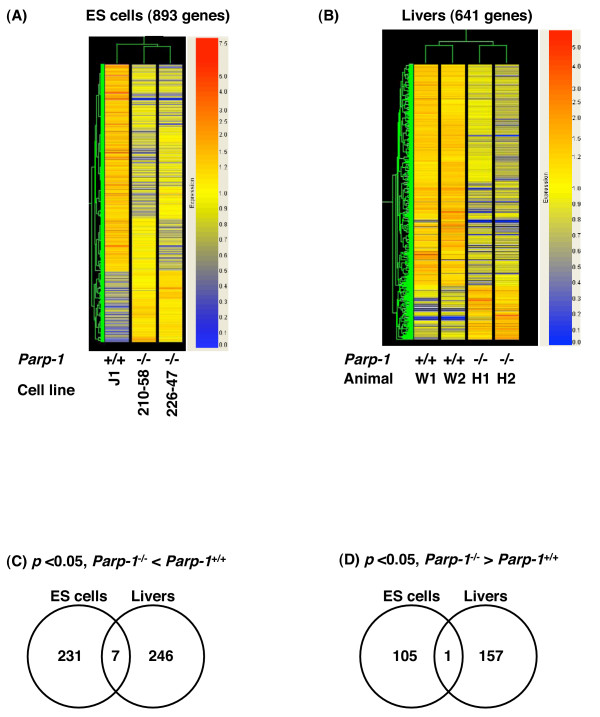
**Comparison of gene expression profiles among cell lines or cell types**. Heatmaps of gene expression profiles in ES cells (A). We constructed the heatmaps using the gene lists containing the genes that showed a difference at *p *< 0.05 in ES cells. Each heatmap is constructed using GeneSpring GX ver. 7.3.1. Numbers of genes down-(C) or up-(D) regulated in common between *Parp-1^-/-^*ES cells and livers. The numbers of the genes are indicated in Venn diagrams. These genes showed the difference with at least 2-fold between *Parp-1^+/+^*and *Parp-1^-/-^*(*p *< 0.05). Fig. 2B in the original article [1] remains unchanged and is presented as (B). Fig. 2D & F in the original article [1] are removed and Fig. 2C & E were corrected in the original article [1] and are presented as (C) and (D).

We also made the heatmaps using the gene lists containing the 893 genes that showed a difference at *p *< 0.05 in ES cells (Fig. 2A (corrected)). Although we used independently isolated *Parp-1^-/- ^*ES cell clones, a clear and common alteration in the gene expression profile was observed (see Fig. 2A (corrected), and Tables 2 (corrected) and 3 (corrected)).

**Table 2:** Genes down-regulated in *Parp-1*^-/- ^ES cells

	**Fold change**^a)^					
				
**Accession No.**	**W vs H**	**J1 vs 210–58**	**J1 vs 226–47**	**Symbol**	**Chromosome**	**Gene description**
**Cell cycle/cell proliferation/cell death**						
AW122355	3.2	5.2	2.3	*Prkcbp1*	2	Protein kinase C binding protein 1
AF067395	2.9	2.9	2.9	*Bnip3l*	14	BCL2/adenovirus E1B 19 kDa-interacting protein
AI842277	2.7	2.3	3.2	*Igfbp3*	11	Insulin-like growth factor binding protein 3
U95826	2.2	2.5	1.9	*Ccng2*	5	Cyclin G2
						
**Cell structure/cell adhesion**						
U16741	4.1	6.3	3.1	*Capza2*	6	Capping protein (actin filament) muscle Z-line, alpha 2
AI132380	3.6	3.1	4.3	*Fndc3a*	14	Fibronectin type III domain containing 3a
AI505453	2.9	2.5	3.4	*Myh9*	15	Myosin, heavy polypeptide 9, non-muscle
AW208938	2.4	3.2	2.0	*Pkp2*	16	Plakophilin 2
M76124	2.4	2.2	2.6	*Tacstd1*	17	Tumor-associated calcium signal transducer 1
						
**Metabolism**						
U73820	5.5	5.2	5.8	*Galnt1*	18	Polypeptide GalNAc transferase-T1 (ppGaNTase-T1)
AI841270	3.4	2.4	6.4	*Gstm1*	3	Glutathione S-transferase, mu1
AV308550	2.6	4.1	1.9	*Piga*	x	Phosphatidylinositol glycan, class A
AI851912	2.3	2.2	2.5	*Rps27*	3	Ribosomal protein S27
AI852144	2.1	2.9	1.7	*Pbef-pending*	12	Pre-B-cell colony-enhancing factor
U65986	2.1	1.9	2.5	*Anxa11*	14	Annexin A11
D50264	2.1	1.4	4.1	*Pigf*	17	Phosphatidylinositol glycan, class F
AF031486	2.0	2.0	2.0	*Sms*	x	Spermidine synthase
AI845882	2.0	2.5	1.7	*Acyp1*	12	Acylphosphatase1, erythrocyte (common) type
						
**Protein biosynthesis/degradation**						
AI852581	3.0	3.0	3.1	*Ide*	19	Insulin degradating enzyme
AI414051	3.0	1.8	9.1	*Usp24*	4	Ubiquitin specific protease 24
AW121012	2.9	2.8	3.0	*Rnf19*	15	Ring finger protein 19
X92665	2.9	2.5	3.4	*Ube2e1*	14	Ubiquitin-conjugating enzyme UbcM3
AW048882	2.2	2.8	1.8	*Iars*	13	Isoleucine-tRNA synthetase
AA867340	2.2	1.9	2.6	*Psme4*	11	Proteasome (prosome, macropain) activator subunit
AB024427	2.2	2.3	2.1	*Rnf11*	4	Ring finger protein 11
						
**Signaling**						
AI846023	4.6	2.8	13.1	*Arl7*	1	ADP-ribosylation factor-like 7
AA260005	2.8	2.7	2.8	*Pawr*	10	PPKC, apoptosis, WT1, regulator
AI317205	2.6	2.4	2.7	*Map3k1*	13	Mitogen activated protein kinase kinase kinase 1
AF035644	2.3	2.0	2.7	*Ptp4a2*	4	Protein tyrosine phosphatase 4a2
M21019	2.3	1.9	2.9	*Rras*	7	Harvey rat sarcoma oncogene, subgroup R
AI194248	2.2	2.5	1.9	*Csnk2a1*	2	Casein kinase II, alpha 1 polypeptide
AI854006	2.0	2.0	2.1	*Set*	2	SET translocation
D83921	2.0	1.9	2.1	*Ebaf*	1	Endometrial bleeding associated factor
						
**Transcription/replication**						
X14206	9.9	8.4	11.9	*Adprt1*	1	Poly(ADP-ribose) polymerase 1
M99167	3.0	6.2	2.0	*Hnrpa1*	15	Heterogeneous nuclear ribonucleoprotein A1
AW107922	2.8	3.7	2.2	*Sox11*	12	SRY-box containing gene 11
AI849135	2.5	2.5	2.5	*Foxo3a*	10	Forkhead box 03a
Y07836	2.5	2.3	2.8	*Bhlhb2*	6	Basic-helix-loop-helix domain containing, class B2
X74760	2.5	2.3	2.7	*Notch3*	17	Notch gene homolog 3, (Drosophila)
AI447783	2.1	2.4	1.9	*Helb*	10	Helicase (DNA) B
X94694	2.1	2.7	1.7	*Tcfap2c*	2	Transcription factor AP-2, gamma
AF077861	2.1	2.2	2.1	*Id2*	12	Inhibitor of DNA binding 2
AI605405	2.0	1.9	2.3	*Phf13*	4	PHD finger protein 13
D78382	2.0	1.7	2.6	*Tob1*	11	Transducer of ErbB2.1
						
**Transport**						
AV356315	4.1	5.5	3.3	*Lman1*	18	Lectin, mannose-binding, 1
AV298789	2.9	2.6	3.2	*Ranbp5*	14	Ran binding protein 5
D88315	2.2	2.2	2.2	*Hiat1*	3	Hippocampus abundant gene transcript 1
						
**Unknown**						
AI845617	3.5	3.5	3.4	*2610019A05Rik*	11	Hypothetical protein
AI852287	3.2	3.3	3.2	*Ankrd28*	14	Ankyrin repeat domain 28
AI836771	3.0	2.8	3.3	*2900008M13Rik*	15	Unknown EST
AA684456	2.9	2.1	4.5	*2310015N07Rik*	7	Hypothetical protein
AI848435	2.8	1.9	4.8	*C78339*	13	Unknown EST
AW123157	2.8	2.5	3.1	*1700051E09Rik*	11	Hypothetical protein
AW124843	2.6	3.1	2.3	*C85108*	4	Unknown EST
AA710439	2.6	2.0	3.6	*6230421P05Rik*	16	Unknown EST
AI853444	2.5	1.8	3.9	*2610042L04Rik*	14	Hypothetical protein
AI853444	2.2	2.1	2.3	*2610042L04Rik*	14	Hypothetical protein
AW121353	2.1	1.6	3.1	*Lrrc8*	2	Luecine rich repeat containing 8
AI037493	2.1	1.5	3.4	*Tbc1d15*	10	TBC1 domain family, member 15
AI461803	2.1	2.2	1.9	*1300006C19Rik*	9	Hypothetical protein
AW049969	2.0	2.0	2.1	*C330005L02Rik*	9	Hypothetical protein
AI847483	2.0	2.0	2.0	*Tmem41b*	7	Transmembrane protein 41B

^a)^W, wild-type cells (J1); H, *Parp-1*^-/- ^ES cells (210–58 and 226–47).

**Table 3:** Genes up-regulated in *Parp-1*^-/- ^ES cells

	Fold change^a)^					
				
Accession No.	H vs W	210–58 vs J1	226–47 vs J1	Symbol	Chromosome	Gene description
**Cell cycle/cell proliferation/cell death**						
X58196	3.1	3.3	2.9	*H19*	7	H19 non-coding RNA
AI842665	3.0	3.1	2.8	*Tax1bp3*	11	Human T-cell leukemia virus type I binding protein 3
						
**Cell structure/cell adhesion**						
X04017	2.3	2.3	2.3	*Sparc*	11	Cysteine-rich glycoprotein SPARC
M26071	2.1	2.5	1.8	*F3*	3	Coagulation factor III
M91236	2.1	2.1	2.1	*Gjb5*	4	Gap junction membrane channel protein beta 5
						
**Immune response**						
U13705	2.3	2.1	2.4	*Gpx3*	11	Glutathione peroxidase 3
						
**Metabolism**						
AW120625	2.3	1.9	2.7	*Pgd*	4	Phosphogluconate dehydrogenase
M64782	2.2	1.9	2.5	*Folr1*	7	Folate-binding protein 1 (FBP1)
X97755	2.0	2.1	2.0	*Ebp*	x	Phenylalkylamine Ca^2+^ antagonist (emopamil) binding protein
						
**Protein biosynthesis/degradation**						
W71352	3.9	4.2	3.6	*Bag2*	1	Bcl2-associated athanogene 2
AI844175	3.4	3.4	3.4	*Mrps11*	7	Mitochondrial ribosomal protein S11
U16163	2.9	2.9	2.8	*P4ha2*	11	Prolyl 4-hydroxylase alpha(II)-subunit
D00622	2.5	2.0	3.0	*Lrpap1*	5	Low density lipoprotein receptor related protein, associated protein 1
X60676	2.3	2.4	2.2	*Serpinh1*	7	HSP47
AW124432	2.1	1.8	2.5	*Mrpl12*	11	Mitochondrial ribosomal protein L12
AI839392	2.0	2.0	2.1	*Aars*	8	Alanyl-tRNA syntase
						
**Transcription/replication**						
D49473	3.4	3.0	3.7	*Sox17*	1	SRY-box containing gene 17
U51335	2.5	2.5	2.6	*Gata6*	18	GATA-binding protein 6
U79962	2.4	2.1	2.6	*Tarbp2*	15	TAR (HIV) RNA binding protein 2
D49473	2.1	1.9	2.3	*Sox17*	1	SRY-box containing gene 17
						
**Transport**						
D14077	2.2	2.1	2.3	*Clu*	14	Clusterin
						
**Others**						
M34603	2.6	2.3	3.0	*Prg*	10	Proteoglycan core protein
AA793009	2.3	2.0	2.7	*Tex19*	11	Testis expressed gene 19
						
**Unknown**						
AI846553	3.2	3.0	3.3	*1110020C13Rik*	15	Hypothetical protein
AI845664	2.1	2.0	2.2	*Grwd*	7	Glutamate-rich WD repeat containing 1

^a)^H, *Parp-1*^-/- ^ES cells (210–58 and 226–47); W, wild-type cells (J1).

We further selected the genes that showed relatively high expression levels (the "Flag value" in GeneSpring ver. 6.1 of the genes should be either "Present" (high level of expression) or "Marginal" (moderate level of expression) in all replicates of the genotype within the 893 genes that showed a difference at *p *< 0.05, see Table 1 (corrected)). Among the 85 genes selected by this analysis, there were 61 genes, obviously including the *Parp-1 *(*Adprt1*) gene itself, that were down-regulated and 24 genes up-regulated, as listed in Tables 2 (corrected) and 3 (corrected).

### Gene expression profile of the livers and EF cells

In the livers, 3.3% (411/12,353) of genes showed a significant difference in expression level (*p *< 0.05) between the *Parp-1 *genotypes. In the livers of *Parp-1^-/-^* mice, 2.0% (253/12,353) of the genes were down-regulated and 1.3% (158/12,353) of the genes were up-regulated (*p *< 0.05). Similar to *Parp-1^-/-^* ES cells, a higher percentage of the genes, 62% (253/411), were down-regulated and the remaining 38% were up-regulated (Fig. 1C–E in the original article [1], and Table 1 (corrected)). The expression of representative marker genes of the liver, including *albumin *(*Alb1*) and *phosphoenolpyruvate carboxykinase *(*Pepck*), was similarly high in both *Parp-1 *genotypes.

The heatmaps were constructed using the gene lists containing the 641 genes that showed a difference at *p *< 0.01 in livers (Fig. 2B). *Parp-1 *deficiency commonly altered gene expression profiles in the livers of two mice analyzed (Fig. 2B, and Table 4 in the original article [1]).

### Comparison of the profiles among different cell types

We compared gene expression profiles between *Parp-1*^-/-^ ES cells and the livers. There were no genes commonly up- or down-regulated as summarized in Tables 2 (corrected), 3 (corrected), and 4 in the original article [1], namely in the genes showing relatively high expression levels selected by Flag values, although we observed that 7 genes, including *Eif2s2 *(*eukaryotic translation initiation factor 2 subunit 2 beta*), *Parp-1*, and 1 gene *Crygs *(*crystallin gamma S*), were commonly down- and up-regulated in the ES cells and livers (*p *< 0.05), respectively (Fig. 2C (corrected) &2D (corrected)).

## Corrected methods in the text

### Data analysis

Data analysis was performed with the GeneSpring^® ^software ver. 6.1 and ver. 7.3.1 (the latest version). For statistical analyses, the fluorescence intensity (raw signal) was normalized to the 50th percentile reading per chip, and then normalized to the median reading per gene. We performed the non-parametric tests with the cross-gene error model being inactive. In the case of *Parp-1*^-/-^ ES cells, 6 replicates consisting of triplicate microarray results from two *Parp-1*^-/-^ ES cell lines were used. We used the triplicate microarray results from the *Parp-1*^+/+^ ES cell line, J1. We excluded genes that showed a standard deviation greater than 2.0 in the normalized data of both genotypes, and we started analysis with 9,640 genes and ESTs for ES cells (Table 1 (corrected)). We constructed gene lists only with the genes that showed statistical differences (*p *< 0.05) and 2-fold or greater differences in normalized expression levels between *Parp-1 *genotypes. To construct heatmaps, we used GeneSpring^® ^GX ver. 7.3.1 (the latest version).

We regret that this error occurred in the phase of generating the data set in our paper may have caused any inconvenience. In the process of making these corrections, the microarray data were submitted to the gene expression database CIBEX [2] with the following accession number: CBX22.
